# On the yielding of a point-defect-rich model crystal under shear: insights from molecular dynamics simulations

**DOI:** 10.1039/d1sm00662b

**Published:** 2021-08-31

**Authors:** Gaurav P. Shrivastav, Gerhard Kahl

**Affiliations:** Institut für Theoretische Physik and Center for Computational Materials Science (CMS), TU Wien Wiedner Hauptstraße 8-10 A-1040 Wien Austria gaurav.shrivastav@tuwien.ac.at gerhard.kahl@tuwien.ac.at

## Abstract

In real crystals and at finite temperatures point defects are inevitable. Under shear their dynamics severely influence the mechanical properties of these crystals, giving rise to non-linear effects, such as ductility. In an effort to elucidate the complex behavior of crystals under plastic deformation it is crucial to explore and to understand the interplay between the timescale related to the equilibrium point-defect diffusion and the shear-induced timescale. Based on extensive non-equilibrium molecular dynamics simulations we present a detailed investigation on the yielding behavior of cluster crystals, an archetypical model for a *defect-rich* crystal: in such a system clusters of overlapping particles occupy the lattice sites of a regular (FCC) structure. In equilibrium particles diffuse *via* site-to-site hopping while maintaining the crystalline structure intact. We investigate these cluster crystals at a fixed density and at different temperatures where the system remains in the FCC structure: temperature allows us to vary the diffusion timescale appropriately. We then expose the crystal to shear, thereby choosing shear rates which cover timescales that are both higher and lower than the equilibrium diffusion timescales. We investigate the macroscopic and microscopic response of our cluster crystal to shear and find that the yielding scenario of such a system does not rely on the diffusion of the particles – it is rather related to the plastic deformation of the underlying crystalline structure. The local bond order parameters and the measurement of local angles between neighboring clusters confirm the cooperative movement of the clusters close to the yield point. Performing complementary, related simulations for an FCC crystal formed by harshly repulsive particles reveals similarities in the yielding behavior between both systems. Still we find that the diffusion of particles does influence characteristic features in the cluster crystal, such as a less prominent increase of order parameters close to the yield point. Our simulations provide for the first time an insight into the role of the diffusion of defects in the yielding behavior of a defect-rich crystal under shear. These observations will thus be helpful in the development of theories for the plastic deformation of defect-rich crystals.

## Abstract 2

The yielding of cluster crystals under shear is primarily governed by the deformation of the underlying (FCC) crystalline structure.

## Graphical abstract

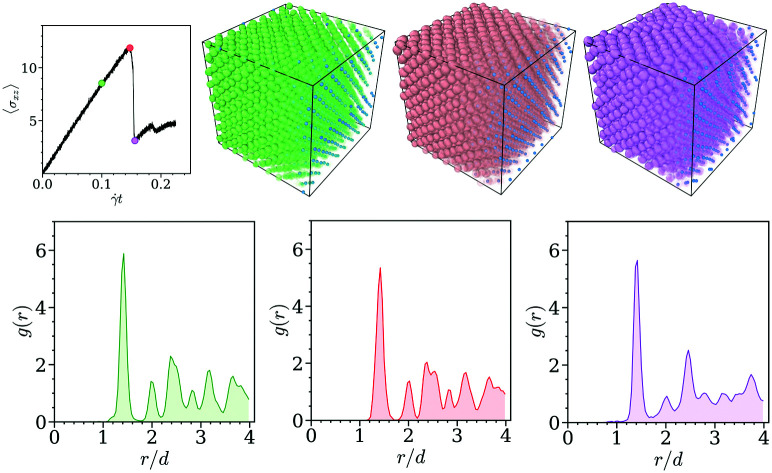

## 1 Introduction

Defects do play a crucial role in determining the mechanical properties of crystals.^1,2^ At finite temperatures real crystals contain various types of such defects, as dislocations, vacancies, or interstitials, to name a few. In particular the impact of dislocations (line defects) on modifying the mechanical response of crystals has been well understood in theory and in experiment (see, *e.g.*, ref. 3–6): for instance, large scale molecular dynamics (MD) simulations have established the connection between interatomic processes and the mesoscopic behavior predicted by *dislocation-dynamics* simulations.^7,8^ In contrast, the impact of point defects (such as interstitials and vacancies) on the mechanical properties of crystals is considerably less explored. To be more specific point defects give rise to the softening of the mechanical response by reducing the yield stress,^9^ and cause the boson peak anomaly at low-frequencies in non-ideal FCC crystals, which, in turn, originate from a local force imbalance on each atom caused by the lack of centrosymmetry.^10,11^ Furthermore, point defects can facilitate dislocation nucleation^12–14^ and induce creep flow in crystalline solids at high temperatures.^15,16^

Continuum elasticity theory considers “ideal crystals” (*i.e.*, without point defects) and explains the rigidity of crystals as a result of a spontaneous breaking of the continuous translational symmetry.^17^ A recently proposed microscopic theory,^18^ developed within the framework of *linear-response theory* and based on correlation functions, incorporates point defects and is able to successfully predict elastic constants, point-defect densities, dispersion relations, *etc.* for non-ideal crystals.^19^ However, what is urgently required is an extension of this theoretical concept beyond this linear regime, which helps to understand phenomena as the yielding behavior and other non-linear effects, such as the transition from brittle to ductile mechanical response, which are often reported in experiments.^20,21^

Crystalline and amorphous solids, when exposed to an external shear deformation, display a transition to the plastic flow.^5,22,23^ The location of the transition point is marked by a maximum in the stress–strain response of the system and the transient response of matter to deformation often shows distinctively different features: (i) ideal crystals often respond to shear *via* the formation of slip planes;^7^ (ii) in contrast, in amorphous solids the solid to liquid-like transition is mediated by the diffusion of particles.^24^ These systems often respond *via* the formation of inhomogeneous flow patterns, also known as shear bands.^22,23,25^ The formation of such band-like regions depends on the preparation history of initial undeformed glassy state and the deformation rate.^26–28^ The transient response of crystals is initiated by dislocation defects which form before the yield point is reached. These defects interact with each other *via* long-range stress fields and move by gliding on slip planes.^2,7^ The collective dynamics of dislocations controls the plasticity of crystals.^29^ In amorphous solids localized plastic events, namely shear transformations are responsible for the plastic deformation.^30^ In contrast to dislocations in crystals, shear transformations are the process of particle rearrangements occurring locally in a small volume.^31^ These zones are identified in experiments and computer simulations by analyzing the local rearrangements of particle neighborhoods.^32–38^

In all these two cases the yielding can be viewed as a time-scale phenomenon, distinguished by the fact that in the former case the viscosity diverges as the shear stress tends to zero.^39,40^ In contrast, in non-ideal crystals which are rich in point defects it is expected that the diffusion of point defects should distinctively alter the yielding behavior of the crystals, which often results in reduction of the yield stress.^9^ In order to understand the interplay of the shear induced and point-defect diffusion timescales in defect-rich crystals under shear it is hence indispensable to focus on this class of crystals. So far, this problem has only been rarely explored in computer simulations: the reasons for that might either be the low concentration of point-defects in hard sphere crystals^41^ or the long-range character of the interactions of point-defects due to strain fields,^42^ which make their collective dynamics slower at high densities. One therefore has to resort to different systems where these restrictions do not apply.

In an effort to elucidate the role of point defects on non-linear phenomena (such as shear) from an alternative route we consider in this contribution *cluster crystals* which are an archetypical representative of defect-rich crystals: these ordered soft matter systems are formed by ultrasoft, purely repulsive particles which are allowed to overlap even at vanishing temperature, subject to a finite energy penalty. Despite their repulsion these particles aggregate at sufficiently high densities and/or sufficiently low temperature into clusters, which, in turn, occupy the sites of BCC or FCC lattices.^43,44^ On a more intuitive level one can understand this particular behaviour that the clusters of these particles can be viewed as harshly repulsive, effective particle aggregates, whose repulsion is typically by a factor ten stronger than the one of the individual particles. Thus – in view of the neighbouring, strongly repulsive clusters an individual ultrasoft particles is not tempted to leave its cluster unless it has a sufficiently high kinetic energy. Furthermore, on a conceptual level both the mechanism why and under which conditions these particles form – despite their mutual repulsion – stable aggregates^45,46^ as well as the complete phase diagram^43,47,48^ are meanwhile well understood. These systems show crystalline phases at sufficiently low temperatures and high densities that are characterized by highly monodisperse clusters of particles occupying BCC or FCC lattice sites.^43,44,47–50^ In contrast, the arrangement of particles inside the clusters remains disordered. In equilibrium, particles vibrate inside clusters and are able to hop at sufficiently high temperatures from one cluster to a neighboring one (at which occasion the cluster size fluctuates). The longtime dynamics of the particles is diffusive^51^ and the distribution of the jump lengths is found to be exponential at short distances. At large distances, this distribution follows a power-law decay, *i.e.*, a behavior reminiscent of Lévy flights.^51^ Most of the investigations of cluster crystals published so far are based on the so-called generalized exponential model with index *n* (GEM-*n*) with *n* > 2,^43,44^ which allows a complete overlap of the particles with a *finite* energy penalty at vanishing interparticle distance.

To round up this short overview we add that recent *out-of-equilibrium* investigations have explore the steady-state behavior of cluster crystals under shear. It is found that these systems show shear-induced fluidization and string formation at high shear rates.^52–54^ However, the yielding behavior of these cluster crystals has not been explored, so far and is therefore poorly understood.

Cluster crystals can be considered as a representative model system for defect-rich crystals as the fluctuating number of particles pertaining to a cluster at the lattice sites corresponds to interstitials of the crystals. In a similar manner, particles can hop from one cluster to the nearby cluster creating thereby a vacancy at the parent lattice site. Of course, within a short time this vacancy will be filled up by another particle arriving from another, neighboring lattice site. In our previous work^55^ we have demonstrated that cluster crystals exhibit an overshoot in their stress–strain response. For a fixed temperature, the height of this overshoot decreases with a decreasing shear rate. The range of temperatures considered in this previous contribution was limited to high temperatures where at equilibrium the long time dynamics is always found to be diffusive. In contrast, in the present contribution, we consider a considerably broader range of temperatures and – using extensive non-equilibrium MD simulations – study the changes in the structure and in the dynamics of cluster crystals under shear. Our work aims to understand the interplay of defect-diffusion and shear-induced timescales. Hence, we shall focus on a sufficiently high temperature where the equilibrium mean-square displacement (MSD) of the particles shows at long times a diffusive behavior. The range of the considered shear rates is such that it covers a wide range of timescales, both large and small, compared to the equilibrium diffusion timescale. Our investigations, which are based both on the MSD data under shear and on an analysis of the centers of mass (COM) of clusters, reveal that in cluster crystals, diffusion of particles is *not* the primary mechanism for stress relaxation: instead it is rather the plastic deformation of the skeleton (the underlying FCC structure), which governs the yielding behavior. Further comparison with related investigations on an FCC crystal formed by particles interacting *via* a purely repulsive, Weeks–Chandler–Andersen (WCA) potential reveals that defects modify the transient response of cluster crystals. However, the overall yielding behavior remains independent of temperature and diffusion of particles.

The rest of the paper is organized as follows: in Section 2, we introduce the model and provide details of the simulations and the related protocols. Results are presented and discussed in Section 3, while the final section contains the summary of results, concluding remarks, and an outlook to related future investigations.

## 2 System and simulation methods

### Simulation of the cluster crystal

In our cluster crystal system particles interact *via* the generalized exponential (GEM-*n*) potential.^43^ Similar as in preceding contributions^43,51^ we set *n* = 4, thus the interaction potential is given by

1 *Φ*^GEM^(*r*) = *ε*exp[−(*r*/*d*)^4^];

here *r* is the distance between two particles, *d* and *ε* set the length- and energy-scales of the model, respectively; in contrast to the usual notation we use the symbol *d* for the range of the interaction, since the conventional symbol *σ* is reserved to denote in this manuscript the stress. We truncate the potential at a distance *r*_c_ = 2.2*d*; *Φ*^GEM^(*r*) is then shifted to zero so that it vanishes from *r*_c_ onwards. Temperature *T*, density *ρ*, and time *t* are measured in units of *k*_B_*T*/*ε*, *ρd*^3^, and 
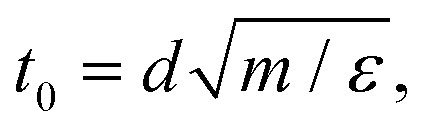
 respectively; further, *m* is the mass of particles and *k*_B_ is the Boltzmann constant. In the following we set the values of *ε*, *d*, *m*, and *k*_B_ equal to unity.

We perform non-equilibrium molecular dynamics (MD) simulations in an *NVT*-ensemble where the number of particles, *N*, the volume, *V*, and the temperature, *T* of the system are fixed. All simulations are carried out using the LAMMPS package.^56^ In this contribution we consider ensembles with *N* = 1300, 3328, 6500, 26 0624, and 52 000 particles; throughout we use a fixed density *ρ* = 6.5 while for the temperature a set of values, namely *T* = 0.4, 0.5, 0.6, and 0.7 has been considered: from literature it is known that at this density and at these temperatures the system assumes a stable FCC cluster phase, where each site of the FCC lattice is occupied by a cluster of overlapping particles (see, for instance, the phase diagram shown in ref. 43). Data available in the literature^43,44,51^ provide evidence that the average number of particles pertaining to a cluster, *N*_c_, assumes for the considered state points a value *N*_c_ ≃ 13 and a lattice constant *l*_a_ = 2. Most of our calculations are based on ensembles of 3328 and 26 624 particles, corresponding thus to systems with 256 and 2048 clusters, respectively.

The temperature of the system is maintained *via* a thermostat, using dissipative particle dynamics (DPD).^57^ The DPD equation-of-motions read (where the dot represents the time-derivative of the respective quantity):2
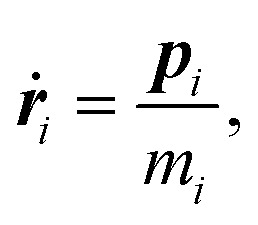
3
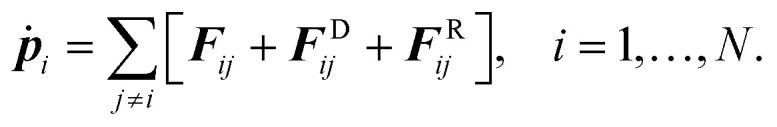
***r***_*i*_ is the position and ***p***_*i*_ is the momentum of the particle with index *i*. The conservative force, ***F***_*ij*_, acting on a pair of particles *i* and *j* can be readily calculated from the interparticle interaction defined in eqn (1). The dissipative force, ***F***^D^_*ij*_, is given by

4 ***F***^D^_*ij*_ = −*ζω*^2^(*r*_*ij*_)(***r̂***_*ij*_·***v***_*ij*_)***r̂***_*ij*_;

***r***_*ij*_ the distance vector between particles *i* and *j*, ***r̂***_*ij*_ is the unit vector of ***r***_*ij*_, and *r*_*ij*_ the distance between the two particles; further, ***v***_*ij*_ = (***v***_*i*_ − ***v***_*j*_) is the relative velocity between particles *i* and *j*, and *ζ* is the friction coefficient; the value of *ζ* is set to unity. Furthermore, *ω*(*r*_*ij*_) is a distance-dependent weight function which defines the range of interaction for the dissipative and random forces. In order to associate the continuous stochastic differential equation with the DPD algorithm^58^ the usual choice for *ω*(*r*_*ij*_) is as follows:^59^5
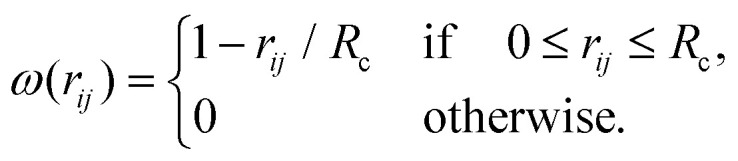


For the cutoff radius of this function, *R*_c_, we have taken for simplicity the same value as for *r*_c_, *i.e.*, *R*_c_ = *r*_c_ = 2.2*d*.

Eventually, the ***F***^R^_*ij*_ represent in eqn (3) random forces, defined as6
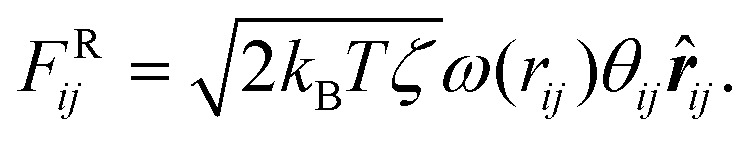


Here, the *θ*_*ij*_ are uniformly-distributed random numbers with zero mean and unit variance. For further details about the parameters of the DPD thermostat we refer to ref. 60 and 61.

The equations-of-motion, *i.e.*, eqn (2) and (3), are integrated *via* the velocity-Verlet algorithm using an integration time step Δ*t* = 0.005*t*_0_.^62^

The initial configurations for our simulations are ideal FCC cluster crystals where each lattice site is occupied by *N*_c_ = 13 completely overlapping particles and assuming a lattice constant that is compatible with the chosen value of the density, *i.e. ρ* = 6.5. Starting from this configuration, the system is equilibrated over 10^6^ MD steps at a temperature *T* = 0.8. The now equilibrated system is further evolved over 5 × 10^6^ MD steps (where it has reached the diffusive regime), storing on a regular basis configurations in intervals of 10^5^ MD steps. These configurations then serve as independent initial configurations for subsequent simulations: from each of these state points, 50 independent simulation runs have been launched. Particle configurations of the system have been stored during the run at logarithmic time intervals; observables were then obtained by averaging the related quantity over these runs.

We impose planar Couette flow on the bulk cluster crystal *via* Lees-Edwards boundary conditions.^63^ The shear is applied in the (*x*,*z*)-plane along the *x*-direction; thus, the *z*- and *y*-directions are the gradient and vorticity directions, respectively, while *x* is the shear-direction. In this study the range of the shear rates, *γ̇*, extends from *γ̇* = 10^−7^ to *γ̇* = 10^−1^ (in units of *t*_0_^−1^).

### 2.1 Simulations on the soft sphere crystal

For the case of soft spheres (SS), which also form an FCC crystals we assumed that the particles interact *via* a Weeks–Chandler–Andersen (WCA) potential, being defined as:^64^7
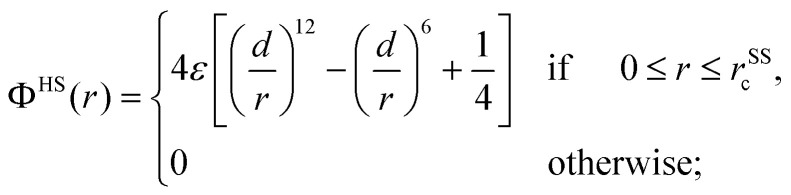
*r* is the distance between two particles, *d* and *ε* are again the length- and the energy-scale of the system; they are both set to unity. The potential is truncated and shifted to zero at a distance *r*^SS^_c_ = 2^1/6^*d*; thus this shifted potential vanishes from *r*^SS^_c_ onwards. For the FCC crystal formed by soft spheres we consider *N* = 4000 particles; for the temperature and the density we assume the following values: *T* = 0.01 and *ρ* = 1.2. Starting from an ideal FCC crystal as an initial configuration we equilibrate the system over 10^7^ MD steps, using again a DPD thermostat (with the same parameters as in the cluster crystal case). We further shear this system with a shear rate *γ̇* = 10^−6^ using the same protocol as for the cluster crystals.

## 3 Results

### 3.1 The system in equilibrium

**Fig. 1:**
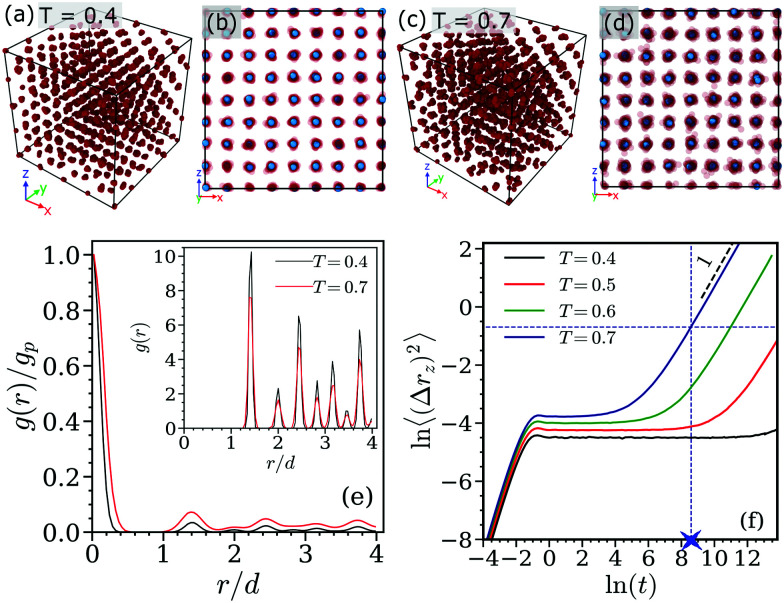
Panels (a) and (c): cluster crystal configurations at equilibrium at temperatures *T* = 0.4 and *T* = 0.7, respectively. Panels (b) and (d): front view of the snapshots shown in panels (a) and (c). Blue spheres represent the centers of mass of clusters. Panel (e): pair correlation function, *g*(*r*), of the clusters as a function of distance, *r*, at temperatures *T* = 0.4 and *T* = 0.7 (as labeled in the inset). The *g*(*r*) are scaled for different temperatures by the value of the first peak, *g*_p_, in the pair correlation functions. The inset shows *g*(*r*) of the centers of mass of the clusters; these positions are highlighted in panels (b) and (d) as blue spheres. Panel (f): *z*-component of the mean-square displacement (MSD) of ultrasoft particles, calculated at different temperatures (as labeled). The horizontal, blue-dashed line indicates where the MSD assumes a value of *d*^2^/2; from the intersection point of this line with the MSD-curve for *T* = 0.7 a vertical, dashed-blue line projects down to the related hopping timescale, *τ*_h_(*T* = 0.7) (see text): *τ*_*h*_(*T* = 0.7) = 5349.44 is marked by blue cross on the time axis. The black dashed line represents a line with slope 1, indicating a diffusive behavior.

In a first step we consider the system in its equilibrium. To this end we briefly summarize the self-assembly and the dynamics of cluster crystals in equilibrium. Panels (a)–(d) of Fig. 1 show simulation generated equilibrium configurations of cluster crystals at a density *ρ* = 6.5 for two different temperatures, namely *T* = 0.4 and *T* = 0.7. The red, semi-transparent, overlapping spheres show the clusters, while the center of mass (COM) of each cluster is plotted as a blue sphere. In panel (e) of Fig. 1 we plot the pair-correlation function, *g*(*r*), of the cluster crystal as a function of distance, *r*, for *T* = 0.4 and *T* = 0.7. The *g*(*r*) are scaled by the value of the first peak, *g*_*p*_, in the pair correlation function. The inset shows the unscaled *g*(*r*) of the COM of the clusters as a function of *r* for the two temperatures. Clearly, the *g*(*r*) of the COM display distinct peaks at distances *r* ∼ 1.41*d* and *r* ∼ 2*d* which are the nearest- and the second-nearest neighbor distances in an FCC crystal with a lattice constant equal to 2*d*. Of course the peaks in the *g*(*r*) broaden at high temperatures indicating the enhanced fluctuations in the COM positions of the clusters.

The *z*-component of the mean-square displacement (MSD) of the individual particles, 〈(Δ*r*_*z*_)^2^〉, is shown for the equilibrium states at temperatures *T* = 0.4, 0.6, and 0.7 in panel (f) of Fig. 1. We only display the *z*-component of the MSD since in our subsequent shear simulations the *z*-axis denotes the gradient direction; thus, 〈(Δ*r*_*z*_)^2^〉 quantifies the non-affine displacement of the particles under shear. Similar to a previous study,^51^ we observe that the MSDs display for all temperatures investigated a short-time, ballistic regime which at later times levels off to a plateau region. At the lower temperature, *i.e.*, at *T* = 0.4, this plateau persists over the entire simulation time window. In contrast, at the higher temperature investigated, *i.e.*, at *T* = 0.7, the MSD shows at larger times a cross-over to a now diffusive regime: this feature originates from hopping processes of individual particles, migrating from one cluster to a neighboring one. In this context it should be emphasized that – despite these hopping events – the underlying FCC structure of the clusters remains intact.^51^ The timescale of these hopping processes, *τ*_h_, can be estimated by identifying the time where 〈(Δ*r*_*z*_)^2^〉 attains a value of *d*^2^/2, corresponding to a distance of half of the nearest neighbor distance in an FCC lattice – note that the nearest neighbor distance in our case is 
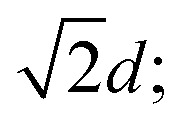
 this value of the MSD is highlighted in panel (f) of Fig. 1 by a horizontal, blue-dashed line. Intersecting this line with the MSD-curve leads – by projection onto the time-axis – to *τ*_h_ = *τ*_h_(*T*); for the temperature *T* = 0.7, *τ*_h_ is highlighted in panel (f) of Fig. 1 by a blue cross. In the following we use the value of *τ*_h_ to classify high and low shear rates in our simulations: introducing the shear-induced timescale, *τ*_s_(=1/*γ̇*) we consider shear rates with *τ*_s_ < *τ*_h_ as high, while shear rates with *τ*_s_ > *τ*_h_ are considered as low.

In the subsequent shear simulations on cluster crystals we focus on two temperatures: (i) *T* = 0.7 for which *τ*_h_ attains a value that is smaller than the total simulation time; therefore we can access for this temperature both the high and the low shear rate windows; (ii) at *T* = 0.4 the value of *τ*_h_ is considerably larger than the accessible total simulation time; hence, at this temperature all shear rates are considered as high.

### 3.2 The system under shear

#### 3.2.1 Stress *vs.* strain response

We start our investigations of the non-equilibrium properties by measuring the stress of the system, *σ*_*xz*_(*t*), as a function of strain, *γ̇t*, for various shear rates, *γ̇*, and at different temperatures. We calculate *σ*_*xz*_*via* the Irving-Kirkwood expression:^65^8
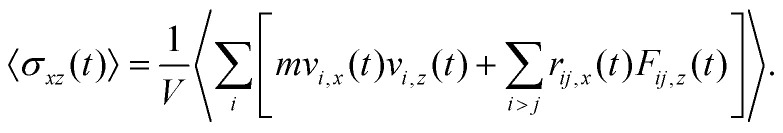


Here, *m* is the mass of the particles, *v*_*i*,*x*_(*t*) and *v*_*i*,*z*_(*t*) represent the *x*- and *z*-components of the velocity of particle *i*; further, *r*_*ij*,*x*_(*t*) is the *x*-component of the displacement vector between particles *i* and *j*, *F*_*ij*,*z*_(*t*) denotes the *z*-component of the force between particles *i* and *j*, and *V* represents the total volume of the system. The angular brackets in eqn (8) stand for an averaging procedure, that is carried out over 50 independent runs (see above). We note that the kinetic terms in eqn (8), *i.e.* the one proportional to *mv*_*i*,*x*_*v*_*i*,*z*_, turn out to be very small; therefore we have neglected them for the calculation of the shear stress.

**Fig. 2:**
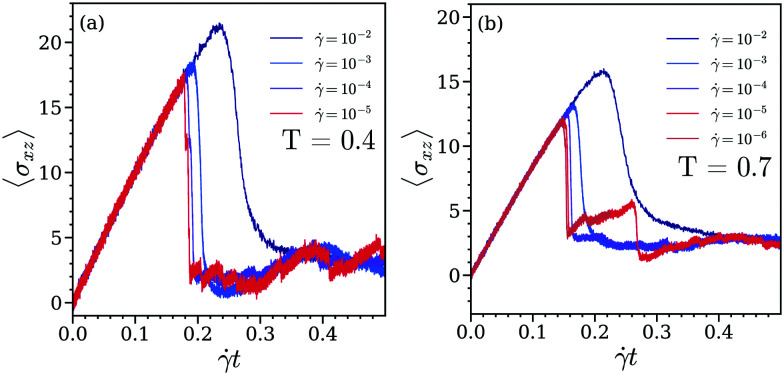
Panels (a) and (b): time evolution of the stress, 〈*σ*_*xz*_〉, for the cluster crystal as a function of strain, *γ̇t*, for different shear rates (as labeled), for the temperatures *T* = 0.4 (a) and *T* = 0.7 (b). Results are based on ensembles of *N* = 26 624 particles.

Panels (a) and (b) in Fig. 2 display the response of the stress on the strain for cluster crystals, calculated at two different temperatures, namely *T* = 0.4 and 0.7; in these calculations ensembles of 26 624 particles were considered. The stress first increases, reaches a maximum and then drops suddenly; this feature corresponds to the yielding of the cluster. We observe that the drop in the stress becomes sharper and that the height of the stress maximum, *σ*_p_, decreases as we decrease the shear rate; these features are observed for all shear rates and temperatures considered. The data shown in panels (a) and (b) of Fig. 2 also provide evidence that for a given shear rate the peak of the stress–strain curve is more pronounced at low temperatures.

**Fig. 3:**
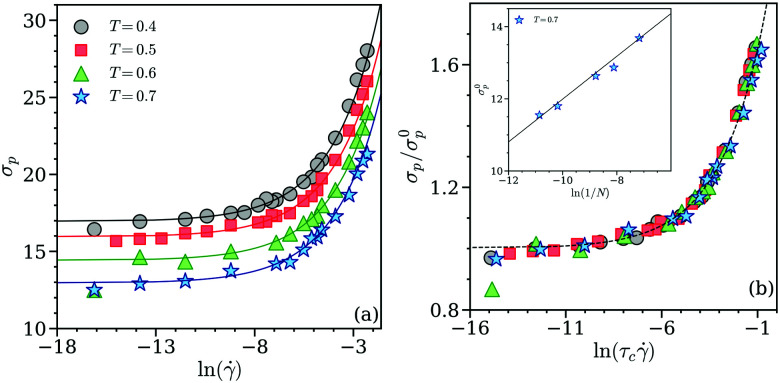
Panel (a): variation of the maximum of the stress–strain curves, *σ*_p_, as a function of the shear rate, *γ̇*, for different temperatures (as labeled). In this panel, the solid lines represent the power-law behavior defined in eqn (9), using suitably fitted parameters *σ*_p_^0^, *A*, and *α*. Panel (b): scaled curves *σ*_p_*vs.* shear rate (shown in panel (a), using eqn (11)); all data collapse on a master curve. The black dashed line represents the scaling function given in eqn (11). Results are based on ensembles of *N* = 3328 particles. The inset in panel (b) shows the variation of *σ*_p_^0^ as function of the system size, considering ensembles of *N* = 1300, 3328, 6500, 26 624 and 52 000 particles at *T* = 0.7. The black solid line represent the logarithmic fit given in eqn (10) (see text).

This feature is further analysed *via* the data shown in panel (a) of Fig. 3 where the maximum of the stress–strain curve is plotted as a function of shear rate for different temperatures on a much finer *γ̇*-grid. We note in passing that these results are now – as a tribute to the high computational costs – based on simulations of ensembles of *N* = 3328 particles; a brief analysis of the data in terms of system size will be given below. High values of *σ*_p_, observed in particular at low temperatures indicate the increased hardness of the cluster crystal; in contrast, at high temperatures the higher diffusivity of particles destabilizes this rigidity, leading to lower values of *σ*_p_. We also note that *σ*_p_ curves, calculated for different values of *γ̇* (as displayed in panel (a) of Fig. 3) can be mapped *via* a power-law function onto a single master curve; this function is given by^23,66^

9 *σ*_p_(*T*,*γ̇*) = *σ*_p_^0^(*T*) + *A*(*T*)*γ̇*^*α*^

with adjustable parameters *σ*_p_^0^, *A*, and *α*. In our considerations the power-law functional form, defined in eqn (9) is similar to the Herschel-Bulkley model used to describe the behavior of the steady-state stress as a function of shear rate.^23^

Fitting the simulation data to the above function leads to the temperature-dependent values of *σ*_p_^0^, *A*, and *α*, which are collected in Table 1. These data are mainly based on investigations of ensembles of *N* = 3328 particles; for two temperatures (*i.e.*, for *T* = 0.4 and *T* = 0.7) we have performed complementary investigations for ensembles of *N* = 26 624 particles, albeit on a sparser *γ̇*-grid. As expected, the values of the fitting parameters, *σ*_p_^0^ and *A*, do show a size dependence, which turns out to be logarithmic in nature. With these parameters at hand we can draw in panel (a) of Fig. 3 the solid lines, using the same color code as the one used for the symbols, representing the simulation data for different temperatures. Our data provide evidence that *σ*_p_^0^ and *A* decrease with increasing temperature, while the exponent *α* (=0.43) remains temperature independent. It seems that the system attains an apparent yield stress, *σ*_p_^0^, *i.e.*, the value of *σ*_p_ at infinitesimally small shear rates. Recent experimental and theoretical studies have revealed that the yield stress decreases with the increase in the system size.^39,40,67,68^ In an effort to analyse our results also in this direction we explore the dependence of *σ*_p_^0^ on the system size. We report that our finite-size analysis of *σ*_p_^0^ provides as a suitable fit a function that decays logarithmically with increasing system size but do not assign too much physical significance to this analysis. The inset in panel (b) of Fig. 3 shows the variation of *σ*_p_^0^ as a function of the inverse of the ensemble size for five different values of *N*, namely *N* = 1300, 3328, 6500, 26 624, and 52 000 at *T* = 0.7. The solid back line represents in this panel a fit of these data with the functional form

10 *σ*_p_^0^ = 17.9227 + 0.5931 ln(1/*N*).

**Table 1:** Parameters *σ*_p_^0^ = *σ*_p_^0^(*T*) and *A* = *A*(*T*) as obtained by fitting the simulation data for *σ*_p_ – see panels (a) and (b) of Fig. 3 – *via* the power-law expression, given in eqn (9); data are listed for the five different temperatures investigated in this contribution. Results are based on investigations with ensemble sizes as indicated (*N* is the number of particles). The temperature-dependent timescale *τ*_c_ is calculated from *A* and *σ*_p_*via τ*_c_ = (*A*/*σ*_p_^0^)^1/*α*^

*T*	*N*	*σ*_p_^0^	*A*	*τ*_c_
0.4	3328	16.9348	28.467	3.3463
0.4	26624	16.8865	24.2684	2.3242
0.5	3328	15.9376	25.4977	2.9827
0.6	3328	14.4088	24.7787	3.5283
0.7	3328	12.9406	24.4795	4.4039
0.7	26624	11.8012	27.967	7.4377

Our data provide at first sight evidence that the yielding (marked by the maximum in the stress–strain response) depends both on the shear rate and temperature. However (and as we will show in the following), the effect of temperature can be scaled out: we learn from panel (a) of Fig. 3 that the *σ*_p_(*γ̇*)–*γ̇* curves obtained for different temperatures show a power-law behavior at large shear rates and saturate to a finite value at low shear rates. Now the fact that the exponent of this power-law at large shear rates remains the same for all temperatures (see above) allows the mapping of these curves onto a single master curve by scaling the shear rate by a temperature-dependent timescale, *τ*_c_(*T*), the latter one being defined as *τ*_c_ = (*A*/*σ*_p_^0^)^1/*α*^; the values of this timescale are listed for different temperatures in Table 1. Starting our reasoning from eqn (9) this master curve has thus the form:^55,69^11
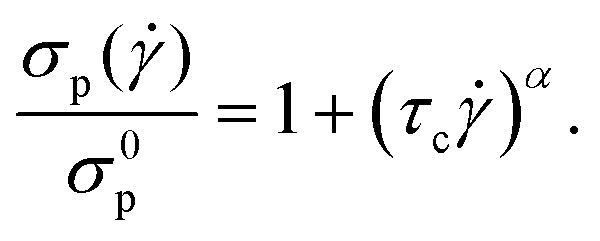


In panel (b) of Fig. 3 we show this master curve onto which the different *σ*_p_(*γ̇*)-curves can be mapped. This obvious scaling behavior provides evidence that in the cluster crystal yielding represents a universal scenario which remains the same for all temperatures; thus yielding is essentially independent of temperature and does not rely on the diffusion of particles.

#### 3.2.2 Dynamics under shear

We further analyze the dynamics of point defects under shear for cluster crystals by focusing on the MSD of our particles. As mentioned in Section 3.1, we consider in the following two different temperatures, *i.e.*, *T* = 0.4 and *T* = 0.7. To obtain information on the non-affine displacement of particles under shear we investigate henceforward in the following the *z*-component of the MSD, 〈(Δ*r*_*z*_)^2^〉, since the *z*-axis represents the direction of the shear gradient in our setup.

**Fig. 4:**
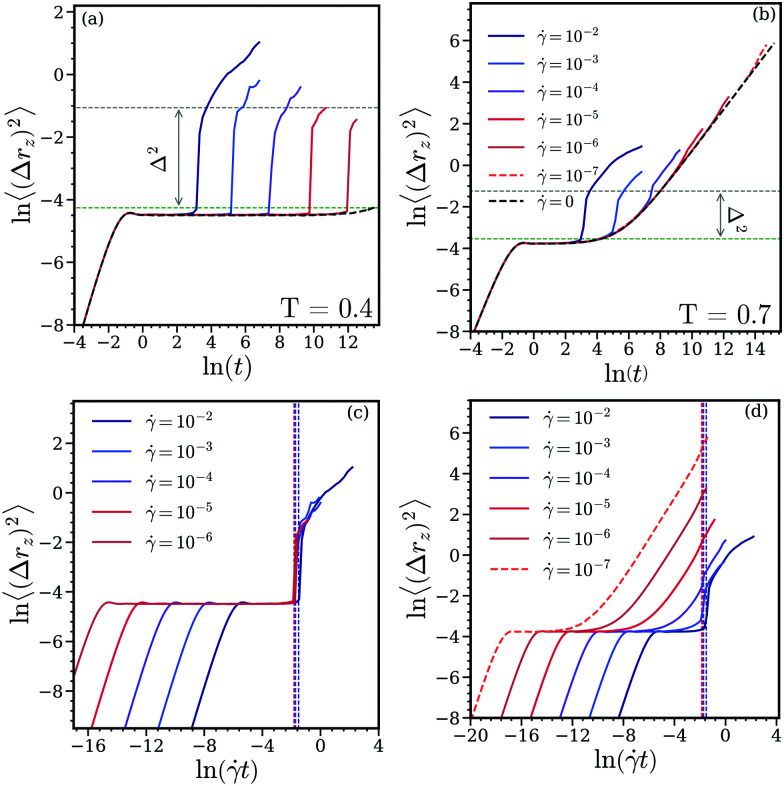
Panels (a) and (b): *z*-component of the MSD, 〈(Δ*r*_*z*_)^2^〉, of a cluster crystal formed by ultrasoft particles in equilibrium and under shear as a function of time *t* at *T* = 0.4 (a) and *T* = 0.7 (b), respectively. The results are shown for six different shear rates, *γ̇* = 10^−2^, 10^−3^, 10^−4^, 10^−5^ and 10^−6^ (as labeled in panel (b)) and for the equilibrium state (*i.e.*, for *γ̇* = 0). Results are based on ensembles of *N* = 26 624 particles except for the MSD of the equilibrium state and for an additional shear rate *γ̇* = 10^−7^ (for *T* = 0.7, in panels (b) and (d)), which are computed from a smaller system with *N* = 3328. These curves are shown by dashed lines in the respective panels. The quantity *Δ*^2^ and the horizontal dashed line are discussed in the text. The green dashed lines represent the values of *R*_*g*_^2^ in both the panels. Panels (c) and (d): *z*-component of the MSD, 〈(Δ*r*_*z*_)^2^〉, of a cluster crystal formed by ultrasoft particles in equilibrium and under shear as functions of the strain, *γ̇t*, at *T* = 0.4 (c) and *T* = 0.7 (d), respectively. The results are shown for six different shear rates, *γ̇* = 10^−2^, 10^−3^, 10^−4^, 10^−5^ and 10^−6^ (as labeled in panel (c)) and for the equilibrium state (*i.e.*, for *γ̇* = 0). Results are based on ensembles of *N* = 26 624 particles. The vertical lines correspond to the maxima in the stress *vs.* strain curves shown in Fig. 2, *i.e.*, they mark the yield strain for the respective shear rate; note that these lines are drawn in the same colours as the respective MSD curves.

Panel (a) of Fig. 4 shows 〈(Δ*r*_*z*_)^2^〉 of the particles at *T* = 0.4, considering different values of the shear rates. In the absence of shear (*i.e.*, for *γ̇* = 0) we observe two distinctively different types of behavior of 〈(Δ*r*_*z*_)^2^〉 as a function of time *t*: first, the expected ballistic regime at short times, which then levels off into a plateau (where the MSD attains a value of ≃10^−2^). Since this plateau persists for *γ̇* = 0 over the entire duration of the simulation (see the black dashed line in this panel) we conclude that at equilibrium essentially no particle hopping occurs. However, as soon as shear sets in we observe four different regimes in the 〈(Δ*r*_*z*_)^2^〉-curves: beyond the ballistic regime (which coincides throughout with the results obtained for the equilibrium case) a plateau occurs where, again, 〈(Δ*r*_*z*_)^2^〉 assumes a value of ≃10^−2^. Now this plateau is of finite length in time *t* and its extent decreases with increasing shear rate *γ̇*. This region is then followed by a pronounced superdiffusive regime, where 〈(Δ*r*_*z*_)^2^〉 changes in an essentially discontinuous manner by nearly one order of magnitude: this feature is – as detailed below – a consequence of the sudden escape of particles from the clusters. The abrupt change in 〈(Δ*r*_*z*_)^2^〉 – delimited in panel (a) of Fig. 4 by the aforementioned plateau and the dashed horizontal line – is denoted by Δ^2^ (see below). At this point it must be mentioned that the onset of the superdiffusive behavior coincides with the yielding point of the stress *vs.* strain curves (see discussion below). In passing we note that such a particular superdiffusive behavior has also been observed in glasses and supercooled liquids under shear, corresponding there to the breaking of neighboring cages of particles.^26,70^ In our system this superdiffusive regime is followed by a smooth increase of the MSD with time; due to the high numerical costs of the simulations we were not able to collect data of sufficient statistical quality in this time window which would allow us to extract the effective exponent of the MSD and to make thereby more detailed conclusions on the nature of this type of particle transport. Returning to the superdiffusive regime, a more quantitative analysis reveals that 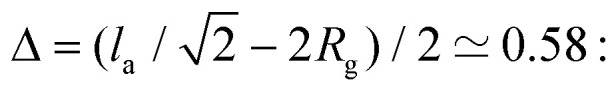 in view of the fact that the nearest neighbor distance between two clusters amounts to 
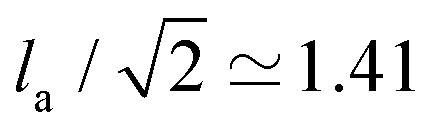
 and estimating the spatial extent of a cluster *via* its radius of gyration, *R*_g_ ≃ 0.12 (its value is denoted by green dashed line in panel (a) of Fig. 4), we conclude that the abrupt change in the MSD must be related to the hopping of the particles from one cluster to a neighboring one. This interpretation is also supported by two additional facts: (i) the actual value of *Δ* is found to be independent of the shear rate; thus we conclude that up to the yielding point particles are either located in clusters or hop from one cluster to another one; (ii) at this low temperature the hopping timescale, *τ*_h_(≪*τ*_s_) assumes an essentially infinite value (see panel (f) of Fig. 1).

The situation is distinctively different at the higher temperature (*T* = 0.7) and possibly more intriguing; the related data are shown in panel (b) of Fig. 4. Again, the MSD shows in the equilibrium state the trivial ballistic behavior at short times, followed by a plateau type region which extends over approximately two orders of magnitude in time; eventually a diffusive process sets in and the MSD grows again with time. Similar as in the low temperature case, the MSD follows under shear initially the related curve of the equilibrium state. Then – depending on the shear rate – the curves of the MSD separate from the equilibrium data *via* a superdiffusive behavior: the higher the shear rate, the earlier the onset of this regime and the more pronounced the superdiffusivity (both in its onset and extent); using the quantity *Δ* introduced above, we observe that *Δ* decreases as the shear rate is lowered. However for low shear rates (*i.e.*, where *τ*_s_ ≫ *τ*_h_) *Δ* is barely noticeable and the transition between the different diffusive regimes becomes rather smooth. Eventually, for *γ̇* = 10^−7^ the MSD curves for the sheared and for the equilibrium state are hardly discernible. This indicates that – although the hopping of particles (or, equivalently the dynamics of the point defects) facilitates stress relaxation – the yielding phenomenon is not entirely associated to the diffusion of the particles.

At this point it should be recalled that cluster crystals are intermediate between liquid-like and crystalline systems: on one side they share features of periodicity of crystals, on the other side they are characterized by liquid-like diffusion of particles and a disordered intra-cluster disordered structure. Therefore, we expect that the yielding of cluster crystals should be based both on the diffusion of particles and on the deformation of the underlying crystalline skeleton. Another evidence for this hypothesis is provided by the fact that the maximum in the stress *vs.* strain curves does not disappear at low shear rates where *τ*_s_ ≫ *τ*_h_.

Eventually we note that the above mentioned features are in striking contrast to non-Newtonian fluids (such as supercooled liquids,^60,61,71^ ferrofluids,^72^ polymers,^73^ mixtures of ferrofluids and liquid crystals,^74^ to name a few) where only diffusion of the particles is responsible for the stress relaxation, and the maximum in the stress *vs.* strain response disappears at low shear rates, *i.e.* where *τ*_*s*_ is comparable to structural relaxation times. Also, at these (low) shear rates, the MSD of the particles under shear coincides with the equilibrium MSD.^60,61^ In contrast, in our cluster crystals, the MSD of particles under shear deviates from the equilibrium MSD at the yielding point even for the lowest shear rate. This suggests that shear induces a deformation of the underlying FCC skeleton, a feature that we shall investigate in more detail in the following sections.

To round up the discussion we now take an alternative view on the data and discuss the MSDs as functions of the strain, *γ̇t*. As mentioned already briefly above, our data show that the onset of the superdiffusive regime coincides at both temperatures considered with the onset of the yield strain (*i.e.*, with the maxima in the stress *vs.* strain curves). Panel (c) of Fig. 4 shows the *z*-component of the MSD as a function of strain for *T* = 0.4 and for the shear rates considered in panel (a) of Fig. 4. The dashed vertical lines mark the yield strains for this temperature, as extracted from the stress *vs.* strain curves (see panel (a) of Fig. 2). We observe that the yield strain weakly depends on the shear rate and that the onset of the superdiffusive regime in the MSD occurs essentially when the cluster crystal yields to the stress. The situations is notably different for the higher temperature, with the related data shown in panel (d) of Fig. 4: again the values of the strain where yielding sets in are weakly dependent on the shear rate. However, we observe two distinctively different scenarios for high (*i.e.*, *γ̇* ≃ 10^−2^ to 10^−3^) and low shear rates: in the former case, the superdiffusive behavior indicates that yielding occurs – similar as for the low temperature – *via* hopping processes from one cluster to the other; in the latter case the smooth variation of the MSDs as functions of strain provide evidence that at lower shear rates yielding is mostly supported by particle diffusion.

In view of the fact that the MSD is a microscopic quantity while the stress–strain response is a macroscopic feature, the above observations suggest that the signatures of yielding at the microscopic scale can be characterized by the displacement of particles from a reference configuration. Such an approach is, for instance, used to identify dynamical heterogeneities at the local scale in glassy systems under shear.^28,70^

Therefore, to characterize the yielding events at the microscopic level we investigate in the following the structural evolution of the cluster crystals under shear using displacements of particles.

#### 3.2.3 Microstructure under shear

We start our analysis at the microscopic level and investigate the displacement field of particles at the two selected temperatures (*i.e.*, *T* = 0.7 and *T* = 0.4) and different values for the shear rate. In the following Figures the non-affine contribution to the displacement field is color coded in two different manners: (i) by the absolute value of its *z*-component (*i.e.*, |Δ*r*_*z*_| – see the color code at the right hand sides of the panels) and (ii) by the direction of the displacement field in the (*z*,*x*)-plane, indicated *via* a green arrow.

**Fig. 5:**
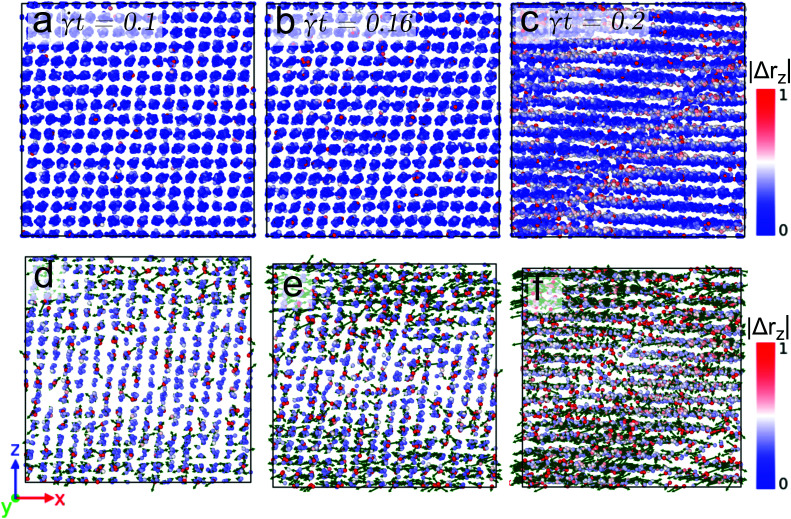
Front view of snapshots of a cluster crystal at strain values *γ̇t* = 0.1 (panel (a)), *γ̇t* = 0.16 (panel (b)), and *γ̇t* = 0.2 (panel (c)) for a temperature *T* = 0.7 and sheared with the rate *γ̇* = 10^−3^. The color axis in all the panels quantifies the non-affine displacement of the particles in the *z*-direction, *i.e.*, |Δ*r*_*z*_|. Panels (d)–(f): highly mobile particles tracked by defining a threshold value on the non-affine displacement, see text. The green arrows indicate the direction of the displacement of highly the mobile particles.

In panels (a) to (c) of Fig. 5 the non-affine displacement of the particles at temperature *T* = 0.7 and for a (relatively high) shear rate of *γ̇* = 10^−3^ is shown. Strain values of *γ̇t* = 0.1, 0.16, and 0.2 have been considered; the corresponding states are located along the strain *vs.* stress curve in the linear regime, near, and beyond the yielding point, respectively (*cf.*Fig. 2). We observe that for the former two cases all the particles have a displacement field that is comparable in size; hence the clusters show a similar extent of displacement. However, beyond the yielding the data of |Δ*r*_*z*_| provide evidence for the formation of slip-lines and that the clusters located along these lines contain particles with high activity. In an effort to elucidate this phenomenon in more detail, we have defined (here and for the following) a threshold value for the non-affine displacement |Δ*r*_*z*_|, namely |Δ*r*_*z*_| = 0.5 before yielding and |Δ*r*_*z*_| = 0.7 beyond yielding in an effort to identify those particles that have propagated over a distance that is equivalent to the nearest neighbor cluster distance. In panels (d) to (f) of Fig. 5 only those highly mobile particles are shown (*via* the above mentioned color code) for the corresponding three strain values – see panels (a) to (c). Our observations can be summarized as follows: (i) with increasing strain the number of mobile particles increases in all clusters, indicating a homogeneous deformation of the cluster crystals up to the yielding point; (ii) at the single cluster level, the mobility of the particles increases in a spatially heterogeneous manner; and (iii) localization of highly mobile particles in the slip-bands occurs.

**Fig. 6:**
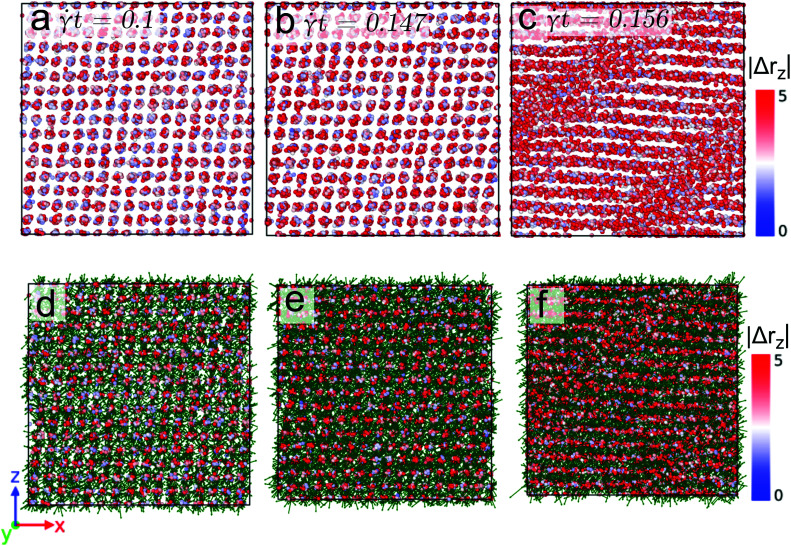
Front view of snapshots of a cluster crystal at strain values *γ̇t* = 0.1 (panel (a)), *γ̇t* = 0.147 (panel (b)), and *γ̇t* = 0.156 (panel (c)) for a temperature *T* = 0.7 and sheared with the rate *γ̇* = 10^−6^. Only the highly mobile particles are shown (see text). The color axis in all the panels quantifies the non-affine displacement of the particles in the *z*-direction, *i.e.*, |Δ*r*_*z*_|. Panels (d)–(f): highly mobile particles tracked by defining a threshold value on the non-affine displacement, see text. The green arrows indicate the direction of the displacement of highly the mobile particles.

We now proceed to a lower shear rate, namely *γ̇* = 10^−6^, but keep the temperature at *T* = 0.7. Here the situation is more intriguing. The related data for the non-affine part of the displacement field of the particles are shown in Fig. 6 where we only display data for the highly active particles (as specified above); panels (a) to (c) show results obtained for states with strain values *γ̇t* = 0.1, 0.147, and 0.156, respectively. Similar as in the case of the high shear rate (see Fig. 5), these state points are chosen in the linear, near, and beyond yielding regime of the stress *vs.* strain curve, see Fig. 2. Note that the values of |Δ*r*_*z*_| now extend over the interval [0, 5] (see color code of Fig. 6). Similar as in the preceding case we observe a homogeneous deformation (below yielding) and the formation of slip-bands (beyond yielding). However, and in contrast to the case of the high shear rate the displacement vectors of the particles (shown in panels (d) to (f) as a green vector) provide evidence of an isotropic movement of the particles at strain values near yielding; this phenomenon is due to the fact that for the low shear rate the timescale of the hopping events of the particles is shorter than the shear-induced timescale. Therefore, particles hop *before* they feel the force due to the external deformation. Eventually after yielding, the displacement of the particles is again oriented along the shear direction.

**Fig. 7:**
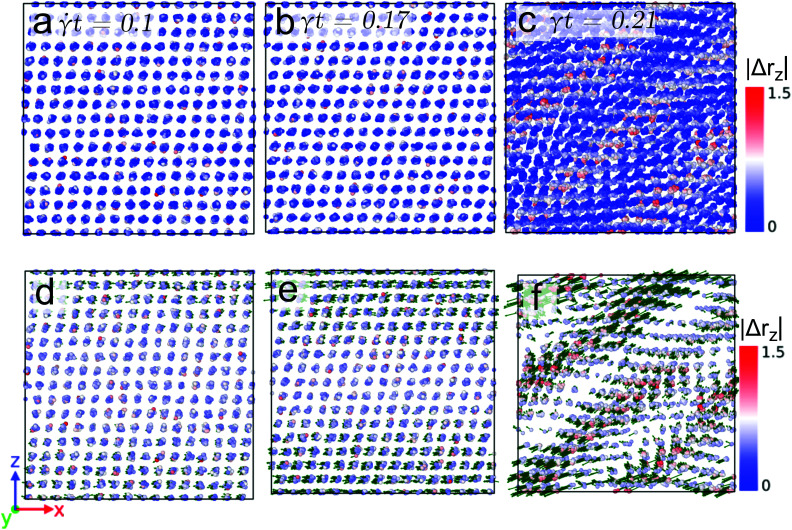
Front view of snapshots of a cluster crystal at strain values *γ̇t* = 0.1 (panel (a)), *γ̇t* = 0.17 (panel (b)), and *γ̇t* = 0.21 (panel (c)) for a temperature *T* = 0.4 and sheared with the rate *γ̇* = 10^−3^. The color axis in all the panels quantifies the non-affine displacement of the particles in the *z*-direction, *i.e.*, |Δ*r*_*z*_|. Panels (d)–(f): highly mobile particles tracked by defining a threshold value on the non-affine displacement, see text. The green arrows indicate the direction of the displacement of highly the mobile particles.

Finally we proceed to the low temperature case (*i.e.*, *T* = 0.4) where the shear-induced effects are more prominent, while the thermally induced disorder is not very pronounced. Fig. 7 shows the non-affine displacement of the particles at *T* = 0.4 and *γ̇* = 10^−3^. The snapshots shown in panels (a) to (c) have been taken from state points with strain values *γ̇t* = 0.1, 0.17, and 0.21, respectively; the state points are again located in the stress *vs.* strain curve before (first two cases) and beyond (latter case) yielding. Note that |Δ*r*_*z*_| now extends from 0 to 1.5. Again and similar to the high temperature case, a homogeneous deformation is observed prior to the yielding and a strongly pronounced post-yielding slip-band formation occurs. The displacement vectors of the highly mobile particles (as defined above), shown in the panels (d)–(f) provide the following evidence: (i) for state points before yielding the orientations of the particles point predominantly along the shear direction; (ii) in striking contrast we observe that beyond yielding the displacement vectors point along the direction of the slip-bands.

The analysis of particle displacements under shear together with MSDs suggest that yielding of cluster crystals involves deformation of the underlying crystalline structure. As already shown by Coslovich *et al.*^51^ particles hop under equilibrium conditions in cluster crystals from one cluster to a neighboring one, while preserving the FCC ordering of the skeleton. Therefore, in an effort to understand the impact of shear on this type of particle transport, we start by identifying the skeleton of the cluster crystal by tracing the centers of mass of the clusters and their time evolution under shear. For this purpose, we focus in the following on the higher temperature (*i.e.*, *T* = 0.7): choosing in the following *γ̇* = 10^−3^ and *γ̇* = 10^−6^ we obtain insight both to the high and the low shear rate regimes (see Section 3.1). We first consider the shear rate *γ̇* = 10^−3^ which represents in our nomenclature the high shear rate regime. We choose one sample of our system and investigate systematically the evolution of the original FCC skeleton as we steadily increase the shear. We note that in cluster crystals it is challenging to assign a unique label to the center of mass of the clusters as clusters keep rearranging with increasing strain. Therefore, tracking the time evolution of the centers of mass or calculating two-time quantities (such as displacement of particles from a reference time) is particularly challenging. In this work, we do not attempt to calculate such quantities for the centers of mass of clusters. Instead, we compare our results at two or more strain points without matching the labels of the center of mass of the clusters.

**Fig. 8:**
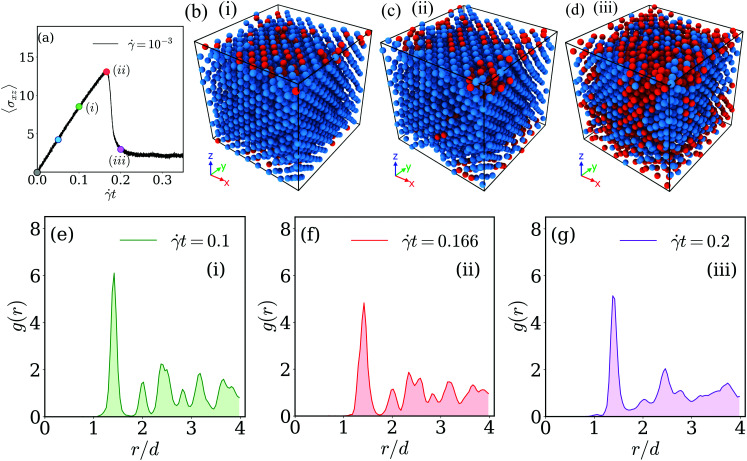
Investigating the skeleton of a cluster crystal under shear – the high shear rate regime (*i.e.*, *γ̇* = 10^−3^). Panel (a): stress *vs.* strain curve for a cluster crystal at *T* = 0.7, exposed to a shear rate of *γ̇* = 10^−3^. Colored circles along this curve indicate selected values of shear (see text); their numbering refers to the labels of the other panels. Panels (b)–(d): snapshot of the skeleton (in terms of the centers of mass of the clusters), shown for three different values of strain ((i) – *γ̇t* = 0.1, (ii) – *γ̇t* = 0.166, and (iii) – *γ̇t* = 0.2): in these snapshots the centers of mass with twelve neighbors are shown in blue, while all other centers of mass are shown in red. Panels (e) – (g): radial distribution functions, *g*(*r*), as functions of the distance, *r*, shown for the very same values of strain as the corresponding snapshots of the skeletons in the panels above. The actual values of strain are shown in the upper right corner of the respective *g*(*r*) panels. Results are based on ensembles of 26 624 particles.

**Fig. 9:**
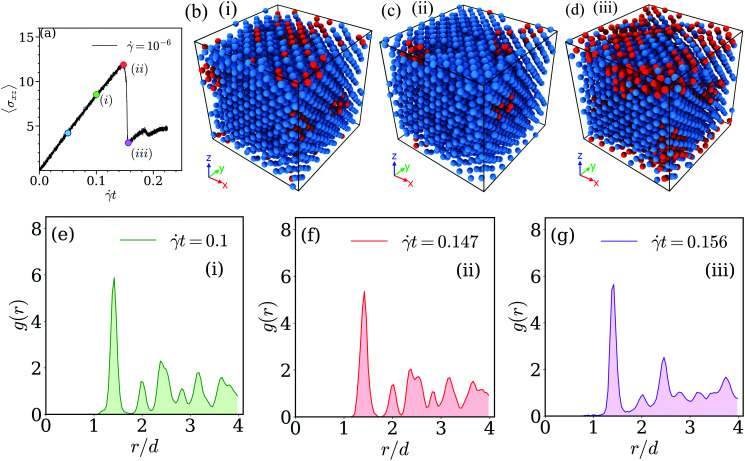
Investigating the skeleton of a cluster crystal under shear – the low shear rate regime. Investigations have been carried out at *T* = 0.7 and at a shear rate *γ̇* = 10^−6^. Same as Fig. 8.

To this end we have marked along the stress *vs.* strain curve (shown in panel (a) of Fig. 8) five values for the strain, each of them being marked by a coloured circle and a numeric label: the first one (coloured in grey) corresponds to the equilibrium case, while the strain values marked in blue and green (the latter one with label (i)) are located in the linear response regime; the fourth value of strain (coloured in red and labeled (ii)) is located at the maximum of the stress *vs.* strain curve, while value (iii) (and colored in purple) has been chosen in the transient regime beyond this maximum. Simulation snapshots corresponding to states (i) to (iii) are shown in panels (b) to (d) of Fig. 8; there the centers of mass of the clusters are coloured according to their immediate vicinity: centers of mass with twelve neighbors are shown in blue, while all other points of the skeleton are colored in red. The respective radial distribution functions are shown in the bottom row of this figure, using the labeling and coloring introduced above. The snapshots and the results for the radial distribution functions suggest that in the linear regime of the stress *vs.* strain curve the FCC structure of the skeleton is marginally affected by the strain (case (i)): only for a few centers of mass the ideal surrounding of twelve neighbors is disturbed while the peaks in the *g*(*r*) are located at the expected positions. However, close to the maximum in the stress *vs.* strain curve (*i.e.*, case (ii)) the peaks of *g*(*r*) broaden and the characteristic pattern of the peak positions is lost; concomitantly a significant fraction of centers of mass has lost its ideal FCC surrounding. Eventually, in the transient regime (case (iii)) the FCC order of the skeleton is almost completely destroyed: the radial distribution function shows a liquid like behavior while a few centers of mass have still preserved their ideal number of twelve nearest neighbors. For the low shear rate, *γ̇* = 10^−6^, we end up with essentially the same conclusions as for the high shear rate case. Related results are now summarized in the panels of Fig. 9: panel (a) shows the stress *vs.* strain curve (defining the five selected values of strain), panels (b) to (d) show the centers of mass of the clusters of a selected simulation snapshot, while panels (e) to (g) show the corresponding radial distribution functions *g*(*r*).

#### 3.2.4 Characterizing the structure at the local scale under shear

Local crystalline order can be conveniently analysed *via* the Steinhardt order parameters or local bond order parameters.^75–78^ In the following we use these parameters to probe the evolution of the local structure of the skeleton of the cluster crystal under shear.

To be more specific we calculate bond order parameters *q̄*_6_ and *q̄*_4_ which are defined as:^76^12
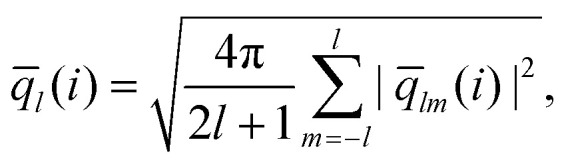
with13
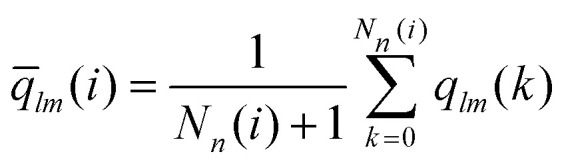
and14
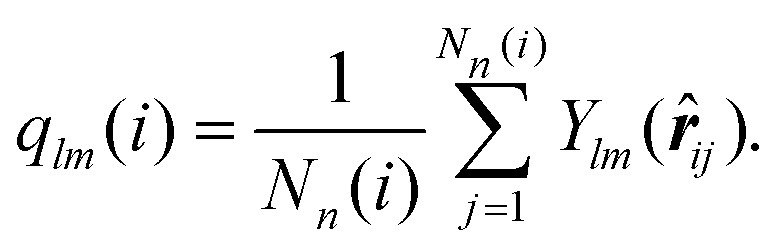


In relation (13) the sum includes all *N*_*n*_(*i*) neighboring clusters of cluster *i* and – *via* the (*k* = 0)-term – the cluster *i* itself. Further, the *Y*_*lm*_(***r̂***_*ij*_) are the spherical harmonics with ***r̂***_*ij*_ being the normalized displacement vector from the center-of-mass of cluster *i* to the one of cluster *j*. Note that the above definition of local bond order parameters involves an additional averaging over the centers-of-mass of the clusters in the surrounding second shell of cluster *i*. At a given strain, we use the distance of the first minimum in the pair correlation function to determine the neighbors of a center of mass of a cluster.

**Fig. 10:**
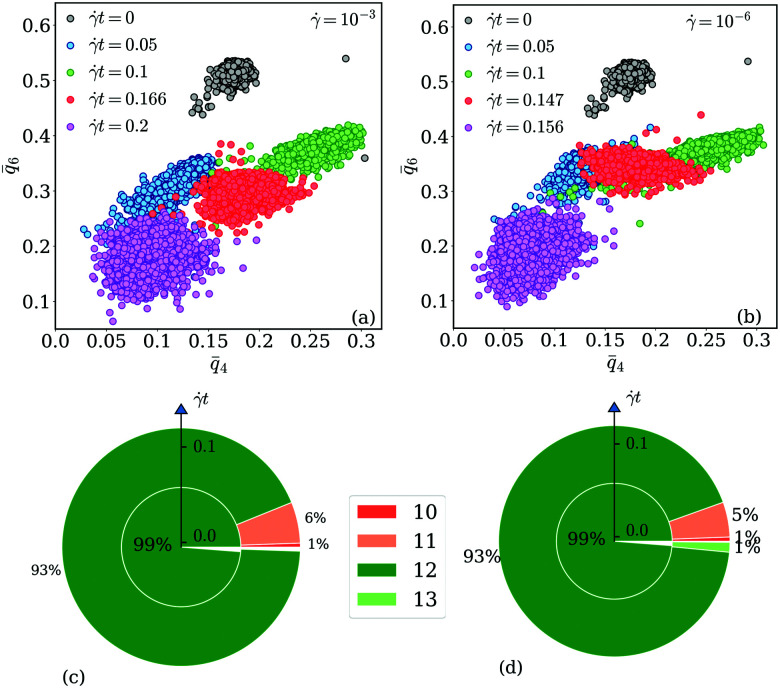
Panel (a): scatter plot of the bond order parameters *q̄*_6_ and *q̄*_4_, defined in eqn (12) for strain values *γ̇t* = 0, 0.05, 0.1, 0.166 and 0.2. The shear rate is fixed to *γ̇* = 10^−3^, further *T* = 0.7. Panel (b): scatter plot of the bond order parameters *q̄*_6_ and *q̄*_4_, defined in eqn (12) for strain values *γ̇t* = 0, 0.05, 0.1, 0.147 and 0.156. The shear rate is fixed to *γ̇* = 10^−6^, further *T* = 0.7. Panels (c) and (d): nested pie plots showing the fraction of centers of mass of clusters with different numbers of neighbors for shear rates *γ̇* = 10^−3^ (panel (c)) and *γ̇* = 10^−6^ (panel (d)). The direction of the arrow points in both panels in the direction of increasing strain. The color code indicates the number of neighbors. Results are based on ensembles of 26 624 particles.

Panels (a) and (b) of Fig. 10 show scatter plots – *q̄*_6_*vs. q̄*_4_ – for a cluster crystal at *T* = 0.7 which is exposed to shear with shear rates *γ̇* = 10^−3^ and *γ̇* = 10^−6^, respectively. Data are plotted for the equilibrium state (*γ̇t* = 0) and for four additional values of strain (as labeled in the panels); the corresponding states are marked in the stress *vs.* strain curves shown for the two shear rates in panels (a) of Fig. 8 and 9, respectively, using the same colour code.

Starting from the equilibrium state (where we observe an essentially ideal FCC order) we find that both *q̄*_6_ and *q̄*_4_ decrease with the increasing strain, indicating a steady decrease in the FCC order. However, in the state close to the yield strain (represented by the red symbols) *q̄*_4_ increases substantially; note that the growth in *q̄*_4_ is more pronounced for the lower shear rate. Beyond the yield strain, both bond order parameters assume – independent of the shear rate – rather small values (purple symbols), providing evidence that the system is now in a disordered state; this disorder seems to be more pronounced for the higher shear rate.

The increase of *q̄*_4_ near the yield point indicates the structural changes that the cluster crystal undergoes at the local level. This feature should also be reflected in the coordination number of the clusters consistent with cubic or square in-planar ordering: the pie plots shown in panels (c) and (d) of Fig. 10 provide estimates for the fraction of cluster centers of mass with different coordination numbers. The arrows in these plots indicate the direction of increasing strain and the color map shows the number of neighbors of a given center of mass. Thus, the inner circle shows the coordination number at *γ̇t* = 0, and the outer shell represents the relative values of the coordination numbers for *γ̇t* = 0.1, *i.e.*, close to the yield point of the respective shear rates. From these data we conclude that – irrespective of the shear rate – close to 93% of the cluster centers of mass have twelve neighbors, indicating that – although the FCC ordering of these centers decreases with increasing strain – a large fraction of the centers maintains even close to the yield point the FCC structure. We also learn that the cubic ordering (which should be characterized by eight neighbors around a center of mass) does not occur. Therefore, we conclude that the above mentioned increase in *q̄*_4_ must be connected to an increase in the in-plane square ordering of the centers of mass.

To investigate this issue further we select among the clusters those which have a *q̄*_4_-value higher than a threshold value (which we specify below) and calculate the average angle that the center of mass of this cluster encloses with its neighbors. These averages are defined as15
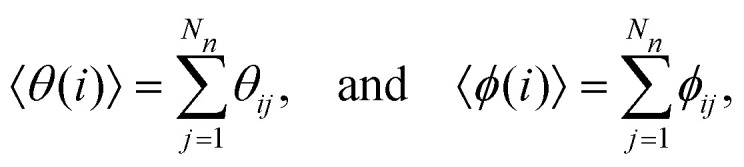
where, *θ*_*ij*_ and *ϕ*_*ij*_ are, respectively, the polar and azimuthal angles (with respect to the coordinate system inherent to the simulation cell) between the center of mass of a tagged cluster *i* and one of its neighbors, carrying the index *j*. The angular brackets denote an average over all the *N*_*n*_ neighboring clusters. It should be noted that in an FCC ordering a cluster has four in-plane neighbors and eight out-of-plane neighbors (*i.e.*, both four in the upper and the lower planes). In the ideal case the values for the in-plane angles are *ϕ* = 45° and *θ* = 90°, while for the out-of-plane case the values of the four angles are given by *ϕ*_1_ = 90°, *θ*_1_ = 45°, *ϕ*_2_ = 0°, and *θ*_2_ = 45°. These values lead to the related average angles 〈*ϕ*〉 = 45° and 〈*θ*〉 = 60° for an ideal FCC structure. We note that the angles are restricted throughout to the first quadrant.

**Fig. 11:**
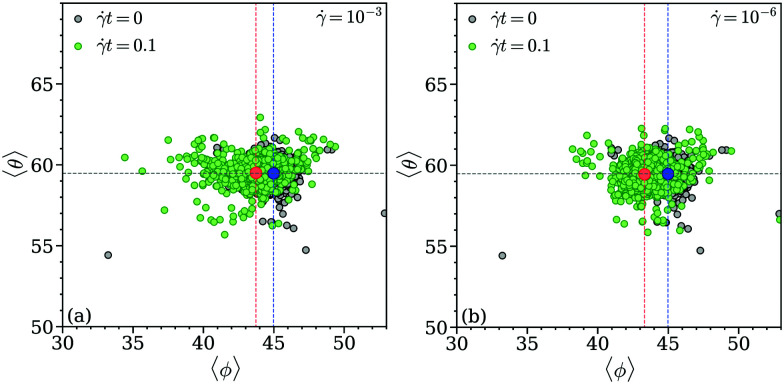
Scatter plots of the average polar 〈*θ*〉 and the average azimuthal 〈*ϕ*〉 angles in a cluster crystal: black symbols – equilibrium state, green symbols – obtained for a strain of *γ̇t* = 0.1; results are based on ensembles of *N* = 26 624 particles for different values of the strain (as labeled). Results are shown for shear rates *γ̇* = 10^−3^ (panel (a)) and *γ̇* = 10^−6^ (panel (b)), respectively. The blue and red circles represent in both the panels the center-of-mass of the black and the green data clouds, respectively. The dashed lines mark the position of the red and the blue symbols in the coordinate system.

For the state where the strain is close to the yield point we choose for the above threshold value *q̄*_4_ > 0.2; this choice is motivated by the fact that in equilibrium (where the FCC order is represented in the most ideal manner), *q̄*_4_ reaches values up to *q̄*_4_ ∼ 0.2 (see panels (a) and (b) in Fig. 10). We note that we have chosen for the equilibrium state clusters with *q̄*_4_ > 0.12 as this value is closer to the lower bound of the scatter plot *q̄*_4_*vs. q̄*_6_ of the equilibrium state; however, this choice has no specific relevance for the following discussion. Panels (a) and (b) of Fig. 11 show the related scatter plots 〈*θ*〉 *vs.* 〈*ϕ*〉 in equilibrium (*γ̇t* = 0) and close to the yield point (*γ̇t* = 0.1) for shear rates *γ̇* = 10^−3^ and 10^−6^, respectively; in addition, the centers-of-mass of these data clouds are highlighted. From the data compiled in Fig. 11 we can make the following conclusions: while we obtain for the equilibrium state the expected values, *i.e.*, 〈*ϕ*〉 = 45° and 〈*θ*〉 = 60°, the value for 〈*ϕ*〉 slightly decreases by ∼3° close to the yield point (irrespective of the shear rate); in contrast, the average polar angle, 〈*θ*〉, remains essentially unaffected by the shear. These findings are a clear indication of a planar shift of clusters along the *x*-direction.

**Fig. 12:**
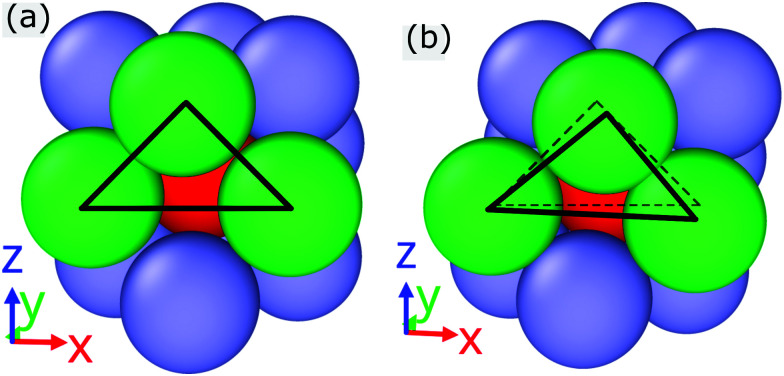
Panel (a): immediate neighborhood of a tagged cluster (red) in the equilibrium state at *T* = 0.7. neighboring clusters are shown in blue and green: the three neighbors located in the forefront (*x*,*z*)-plane are marked in green and are connected by solid black lines. Panel (b): immediate neighborhood of a tagged cluster (red) with *q̄*_4_ = 0.2234, as obtained under shear at a strain *γ̇t* = 0.147 (the shear rate is *γ̇* = 10^−6^). The color coding of the spheres is same as the one in panel (a). The solid black lines connect the green spheres while the dashed black lines represent the related configuration of green spheres in the equilibrium state.

To better understand this issue we choose one cluster with large *q̄*_4_-value and analyze the arrangement of its neighboring clusters. Panels (a) and (b) of Fig. 12 show the positions of neighboring clusters around a tagged cluster equilibrium and for a cluster (with *q̄*_4_ = 0.2234) at *γ̇t* = 0.147 (obtained for a shear rate *γ̇* = 10^−6^), respectively. In both cases, the central cluster is shown in red while its neighboring clusters are coloured in green and blue: the three neighbors located on the foreground (*x*,*z*)-plane are highlighted in green. In the equilibrium state, the green-coloured clusters are arranged at the edges of an equilateral triangle; at the yield point, however, one can notice a substantial shift of the positions of the green clusters along the positive *x*-direction: this is visualized in panel (b) of Fig. 12, where the dashed triangle connects the positions of the cluster in equilibrium. This finding suggests that a shift of the clusters takes place in the (*x*,*z*)-plane along the *x*-direction. Experiments and computer simulations on soft colloidal crystals in two dimensions reveal that stress relaxation involves a highly cooperative movement of particles in the system,^79^ meaning that – while the positions of the particles remain fixed within a plane – the entire planes are shifted. Therefore we also expect a cooperative motion of clusters in the (*x*,*z*)-plane along the shear direction. In an effort to further analyse such a cooperative movement of the clusters, we select at a given value of the strain an (*x*,*z*)-planes and tag one cluster: we then calculate the angles enclosed by the bonds of this cluster with its neighbors which are located in the layer above the tagged cluster.

**Fig. 13:**
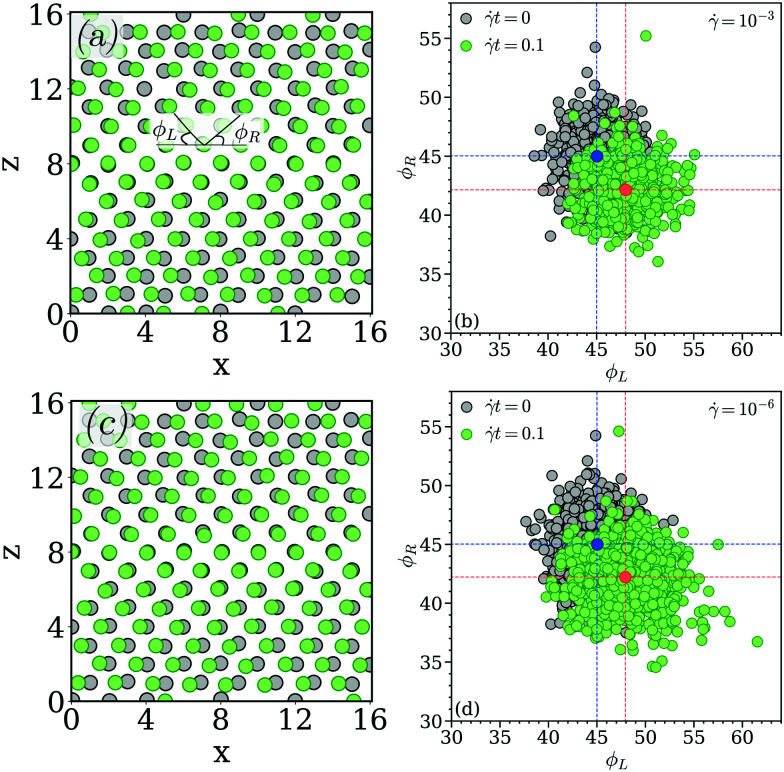
Panel (a): centers of mass of the clusters in the (*x*,*z*)-plane in the equilibrium state (black circles) and at strain *γ̇t* = 0.1 (green circles) for a shear rate *γ̇* = 10^−3^. The solid black lines enclose the planar angels *ϕ*_*L*_ and *ϕ*_*R*_. Panel (b): scatter plot of the planar angles *ϕ*_*L*_ and *ϕ*_*R*_ for a shear rate *γ̇* = 10^−3^ at values of the strain of *γ̇t* = 0 and 0.1 (as labeled). Panel (c): center of mass of the clusters in the (*x*,*z*)-plane in the equilibrium state (black circles) and at strain *γ̇t* = 0.1 (green circles) for a shear rate *γ̇* = 10^−6^. Panel (d): scatter plot of the planar angles *ϕ*_*L*_ and *ϕ*_*R*_ for shear rate *γ̇* = 10^−6^ at values of strain *γ̇t* = 0 and 0.1 (as labeled). In panels (b) and (d) the blue and red circles represent the centres of mass of the data clouds for values of strain *γ̇t* = 0 and 0.1, respectively. The blue and red dashed lines mark the angles *ϕ*_*L*_ and *ϕ*_*R*_ of the centers of mass of the respective data clouds.

Under shear different (*x*,*y*)-planes are shifted in a cooperative manner along the *x*-direction; consequently an (*x*,*z*)-plane corresponds to a vertical cross-section of all these moving (*x*,*y*)-layers for a given *y*. Two such representative (*x*,*z*)-planes, selected at some *y*-position, are shown in panels (a) and (c) of Fig. 13 for the two considered shear rates. The angles *ϕ*_*L*_ and *ϕ*_*R*_ formed by the bonds of the tagged cluster with its nearest neighbors in the layer above are highlighted in panel (a) of Fig. 13. Now we calculate these angle for all clusters and plot the data in a scatter plot, displayed in panels (b) and (d) of Fig. 13 for shear rates, *γ̇* = 10^−3^ and 10^−6^, respectively; the vertical and horizontal lines indicate (along with the red and blue circles) the “centers-of-mass” of these data clouds. Clearly, in the equilibrium state, both the angles attain values close to 45°. However, near the yield point, *i.e.*, at *γ̇t* = 0.1, *ϕ*_*R*_ decreases by ∼3° while *ϕ*_*L*_ increases by the same amount, indicating the cooperative planar movement of the clusters in different layers along the *x*-direction.

#### 3.2.5 Comparison with the soft sphere FCC crystal under shear

The FCC structure of cluster crystals at high density is the consequence of the fact that the densely packed clusters formed by the ultrasoft particles can essentially be considered as soft spheres. To round up our considerations we repeat the preceding analysis and apply it to a system of soft spheres, with interactions specified in eqn (7) in Subsection 2.1. The ensemble consists of *N* = 4000 particles; we consider a temperature of *T* = 0.01 and a density *ρ* = 1.2. For these parameters the systems forms in equilibrium an FCC crystal. Due to the finite temperature the crystal contains defects, whose concentration can be considered to be small: previous work by Bennett and Alder^80^ and by Pronk and Frenkel^41^ provide an estimate of the point defect concentration in a hard sphere FCC crystal, which is of the order of 10^−8^. Even though our system is – due to the 1/*r*^12^-decay of the repulsion – softer than pure hard spheres, we do not expect in our system a considerably higher defect concentration than the value given above. In addition, the low temperature ensures that in equilibrium the defect concentration in the HS systems remains negligible as compared to the cluster crystals.

**Fig. 14:**
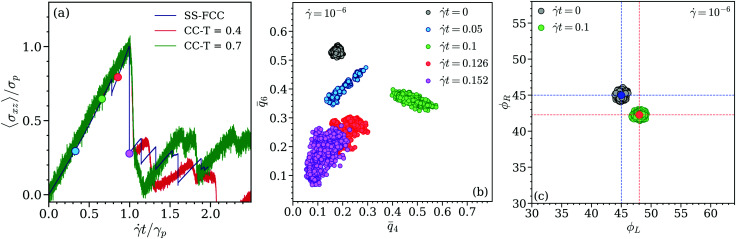
Panel (a): scaled stress, *σ*_*xz*_/*σ*_p_, as a function of scaled strain, *γ̇t*/*γ*_p_, where *γ*_p_ is the strain where the respective system yields at the shear rate *γ̇*. Data are shown for a soft sphere FCC crystal (label SS-FCC) and for cluster crystals (label CC) at *T* = 0.4 and 0.7. All systems have been exposed to shear with a shear rate *γ̇* = 10^−6^. Colored circles mark states along the stress–strain curve that are discussed in the text and the other panels. Panel (b): scatter plot of the averaged local bond order parameters, *q̄*_6_ and *q̄*_4_ for the FCC system formed by soft spheres, for a shear rate *γ̇* = 10^−6^ at values of strain *γ̇t* = 0, 0.05, 0.1, 0.13, 0.152 (as labeled). Panel (c): scatter plot of the planar angles, *ϕ*_*L*_ and *ϕ*_*R*_, for the FCC system formed by soft spheres for a shear rate *γ̇* = 10^−6^ at values of strains, *γ̇t* = 0 and *γ̇t* = 0.1 (as labeled). The blue and red circles in panel (c) represent the centre of mass of the data clouds for values of strain *γ̇t* = 0 and 0.1, respectively. The blue and red dashed lines mark the angles *ϕ*_*L*_ and *ϕ*_*R*_ of the centers of mass of the respective data clouds.

We now shear this system with the shear rate *γ̇* = 10^−6^, applying the same shearing protocol as for the cluster crystals. The stress *vs.* strain response of the soft sphere system, together with the related data of the cluster crystals are compared in panel (a) of Fig. 14: in an effort to superpose these curves we have scaled the stress by its value at the peak and the strain by the yield strain value. For strain values up to the yielding point one can observe a similar response of the two systems.

Further, the local bond order parameters, *q̄*_6_ and *q̄*_4_, shown in panel (b) of Fig. 14 as a scatter plot display a trend similar to the one observed in cluster crystals (*cf.* panels (a) and (b) in Fig. 10). The only remarkable difference is related to the fact that at *γ̇t* = 0.1 (*i.e.*, close to the stress maximum) *q̄*_4_ attains values that are considerably higher than in cluster crystals. As expected, this feature is related to the cooperative planar movement of particles which can be traced *via* the planer angles *ϕ*_*L*_ and *ϕ*_*R*_, defined above for the cluster crystal. Panel (c) of Fig. 14 shows a scatter plot of the angles *ϕ*_*L*_ and *ϕ*_*R*_, both in equilibrium and as well as under shear with *γ̇t* = 0.1 (*i.e.*, close to the yield point). Again, in equilibrium both angles are distributed around 45° (again, the center of the data cloud is highlighted by a blue circle). However, close to the yield point, at *γ̇t* = 0.1, *ϕ*_*L*_ increases by ∼3° and *ϕ*_*R*_ decreases by the same amount, an observation which is consistent with our findings for the cluster crystal (*cf.* panels (b) and (d) of Fig. 13). Due to the fact that in a crystal formed by soft spheres each lattice position is occupied by one single particle, while in a cluster crystal these positions are occupied by a cluster of ultrasoft particles which oscillate around these positions, the data clouds are more compact in the this case.

Summarizing we find that the yielding behavior of the cluster crystals is very similar to the one of FCC crystals, except that (i) the increase in the *q̄*_4_ and (ii) changes in the planar angles are under shear less pronounced; this might be the consequence of diffusing point defects, indicating that the diffusion of particles (or equivalently, of point defects) should have a strong impact on the transient behavior of the cluster crystals and facilitating there by a shear-induced flow in these systems.

## 4 Summary and outlook

In this contribution we have investigated the yielding of an archetypical model of a defect-rich crystal, a so-called cluster crystal; in such a system the particles interact *via* an ultrasoft (*i.e.*, bounded and repulsive) potential and form stable clusters of overlapping particles which populate the lattice sites of an FCC crystal. With the particles incessantly moving from one cluster to a neighboring one such a cluster crystal represents a fascinating system: particles keep diffusing, while the FCC ordering of the system is still maintained. In this sense such crystals can be considered with respect to their vacancy population “intermediate between” a hard sphere crystal (with extremely few defects) and an amorphous solid.

Our results are based on computer simulations with fairly large ensembles (involving up to 52 000 particles) under *NVT* conditions and using a DPD thermostat. Based on existing results, temperature and density have been chosen such that each cluster contains on the average 13 particles. Planar Couette shear is imposed along the *x*-direction on the system *via* Lees-Edwards boundary conditions. Shear rates *γ̇* (measured in internal time units) range from *γ̇* = 10^−7^ to 10^−1^. Two different values for the temperature have been considered: (i) a rather low one where particles remain essentially localized in their cluster: here the characteristic hopping (or diffusion) time *τ*_h_, is considerably larger than the accessible simulation time; (ii) a high value where particles are allowed to diffuse: now *τ*_h_ is comparable to the total simulation time and we can access both high (*i.e.*, 1/*γ̇* < *τ*_h_) and low (*i.e.*, 1/*γ̇* > *τ*_h_) shear rate regimes. The main focus of the work is to understand the role of the diffusion timescale of particles on the yielding behavior of the system under shear.

Irrespective of the temperature we find that throughout the stress–strain curves have a very similar shape. The values of yield stress (*i.e.*, the maximum in these stress–strain curves) obtained for different temperatures and shear rates can be mapped as functions of *γ̇* with remarkable accuracy onto a single master curve for which we have assumed a power-law function with a temperature independent exponent. This result confirms that the macroscopic yielding behavior of cluster crystals remains independent of temperature. At the microscopic level we have systematically investigated the mean square displacement: at low temperatures the cluster crystal reacts on shear *via* a superdiffusive behavior of the particles, which essentially jump at a shear-independent value of strain from one cluster to a neighboring one. At the higher temperature this feature occurs only at rather large shear rates, while at lower shear rates the transition between the ballistic and the diffusive regime is rather smooth. We note that the thereby the diffusion is enhanced, even though the shear-induced timescales are much larger than the equilibrium diffusion timescales. The analysis of the non-affine displacement of particles (in combination with the data of the MSD presented in Fig. 4) indicates that the yielding of cluster crystals is not entirely related to the simple diffusion of particles; it rather involves the deformation of the underlying crystalline structure, which can be characterized by centers of mass of the clusters. Identifying and tracing the centers of mass of the clusters we demonstrate (on the basis of bond order parameters *q̄*_4_ and *q̄*_6_) that the deformation of cluster crystals under shear induces the yielding of the underlying FCC structure. Plastic deformation of the crystal results in the modification of the internal characteristic length scale. From a closer analysis of these quantities along the strain–stress curve we conclude that the reaction of the cluster crystal on shear is related to a cooperative movement of clusters in different layers along the shear direction. This behavior is reminiscent of FCC crystals formed by particles with strongly repulsive interactions: to confirm this conjecture on a quantitative level we have performed complementary investigations on an FCC crystal where particles interact *via* the (repulsive) Weeks-Chandler-Andersen potential and observe similar findings. We conclude that the clusters of a cluster crystal can essentially be viewed as “effective”, soft and repulsive spheres.

With all this in mind our MD simulations reveal that the yielding of cluster crystals depends on the deformation of the underlying FCC structure, while the diffusion of individual particles essentially does not affect the yielding scenario. Further we can conclude that the yielding scenario in cluster crystals is similar to the one of a soft-sphere FCC crystal, which primarily involves the deformation of the underlying crystalline structure.

We expect that the main results of our contribution – *i.e*., that the diffusion of particles is not the primary mechanism of stress relaxation in cluster crystals, but rather the deformation of the underlying FCC structure which is responsible for the stress relaxation – will remain valid for defect-rich crystals. This finding would then induce that topological defects (such as dislocations) do play an essential role in the yielding of such crystals. Therefore, it will be interesting to compare our results with the predictions of the recently proposed microscopic theory for the deformation of defect-free crystals as a diffusion of point defects does not alter the yielding scenario of soft crystals.^40,81^ Furthermore, this indicates the necessity of identifying elementary plastic events at the microscopic level, which further involves the characterization of particle rearrangements. In cluster crystals, such a characterization of defects includes identifying: (i) the shear transformation zones in the clusters, which contain disordered arrangement of particles and (ii) dislocation defects in the underlying crystalline structure of the COMs of clusters.

We note that the component of the MSD of particles in the gradient direction which quantifies the non-affine displacement of particles provides the information about particle displacements and indicates that the yielding event is associated with the hopping of particles. Our results demonstrate that the diffusion of point-defects strongly affects the yield stress and the transient behavior of crystals under shear. However, the characterization of dislocation defects in the underline crystal structure and local rearrangement of particles inside a cluster is urgently required. Such studies are often performed using quasistatic shear protocol under athermal conditions in order to avoid thermal disorder; this will be the topic of subsequent investigations.

## Conflicts of interest

Authors have no conflicts to declare.

## Supplementary Material
